# First genomic resource for an endangered neotropical mega-herbivore: the complete mitochondrial genome of the forest-dweller (Baird’s) tapir (*Tapirus bairdii*)

**DOI:** 10.7717/peerj.13440

**Published:** 2022-06-01

**Authors:** Caroline C. Ennis, Jorge Ortega, J. Antonio Baeza

**Affiliations:** 1 Biological Sciences, Clemson University, Clemson, SC, United States of America; 2 Escuela Nacional de Ciencias Biológicas, Instituto Politécnico Nacional, Ciudad de Mexico, Mexico DF, Mexico; 3 Departamento de Biologia Marina, Universidad Catolica del Norte, Coquimbo, IV Region, Chile; 4 Smithsonian Marine Station at Fort Pierce, Smithsonian Institute, Fort Pierce, FL, United States of America

**Keywords:** Purifying selection, Mitophylogenomics, Phylogenomics, Conservation

## Abstract

Baird’s tapir, or the Central American Tapir *Tapirus bairdii* (family Tapiridae), is one of the largest mammals native to the forests and wetlands of southern North America and Central America, and is categorized as ‘endangered’ on the 2014 IUCN Red List of Threatened Species. This study reports, for the first time, the complete mitochondrial genome of *T. bairdii* and examines the phylogenetic position of *T. bairdii* amongst closely related species in the same family and order to which it belongs using mitochondrial protein-coding genes (PCG’s). The circular, double-stranded, A-T rich mitochondrial genome of *T. bairdii* is 16,697 bp in length consisting of 13 protein-coding genes (PCG’s), two ribosomal RNA genes (*rrnS* (*12s* ribosomal RNA and* rrnL* (*16s* ribosomal RNA)), and 22 transfer RNA (tRNA) genes. A 33 bp long region was identified to be the origin of replication for the light strand (O_L_), and a 1,247 bp long control region (CR) contains the origin of replication for the heavy strand (O_H_). A majority of the PCG’s and tRNA genes are encoded on the positive, or heavy, strand. The gene order in *T. baiirdi* is identical to that of *T. indicus* and *T. terrestris*, the only two other species of extant tapirs with assembled mitochondrial genomes. An analysis of Ka/Ks ratios for all the PCG’s show values <1, suggesting that all these PCGs experience strong purifying selection. A maximum-likelihood phylogenetic analysis supports the monophyly of the genus *Tapirus* and the order Perissodactyla. The complete annotation and analysis of the mitochondrial genome of *T. bairdii* will contribute to a better understanding of the population genomic diversity and structure of this species, and it will assist in the conservation and protection of its dwindling populations.

## Introduction

**Figure 1:**
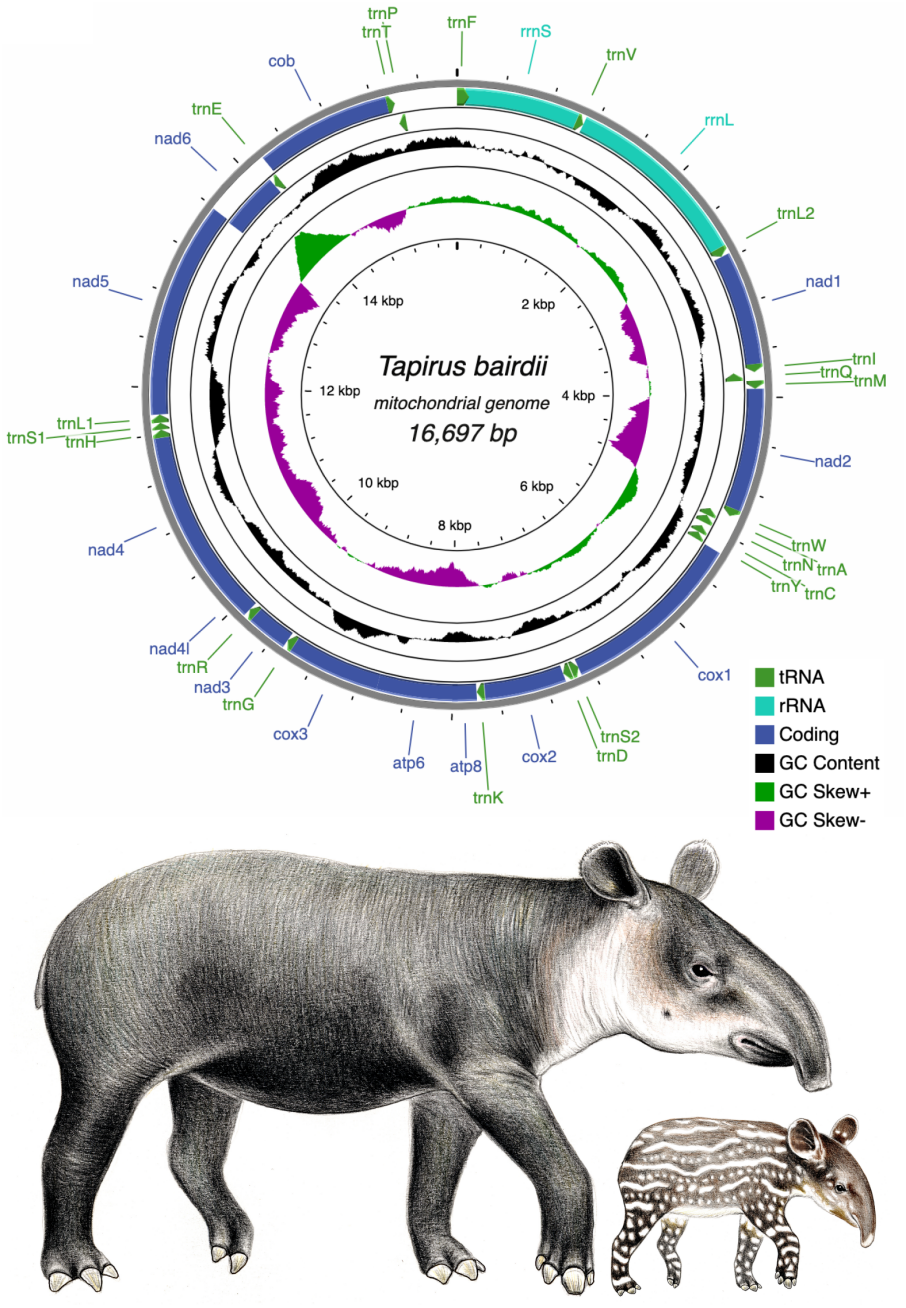
Circular genome map of the mitochondrial DNA of *Tapirus bairdii*. The mitochondrial genome is comprised of 13 protein coding genes (PCGs), 22 transfer RNA genes (tRNA), and 2 ribosomal RNA genes (rRNA). The inner circles show the GC content and GC skew along the sequence. Illustrations of *Tapirus bairdii* copyright 1990 Stephen D. Nash. Used with permission.

In the family Tapiridae, Baird’s tapir (*Tapirus bairdii*), also known as the Central American or Mesoamerica tapir, is the largest mammal native to southern North America and Central America (Fig. 1) and is one of four extant species within the genus *Tapirus* among odd-toed ungulates (order Perissodactyla). *Tapirus bairdii* can be found in forests and wetlands ranging from southern Mexico to Colombia (Schank et al., 2020). They play an important role as seed dispersers in tropical forests, including areas highly threatened by disturbance (Paolucci et al., 2019), and represent a food source for rural-dwelling people (Foerster & Vaughan, 2006). Baird’s tapir browses the forest in areas with nearby freshwater bodies, high tree and shrub diversity that provide good food quality, low predation (including hunting) pressure, and limited human presence (Naranjo, 2009). Humans remain the primary predator of Baird’s Tapir, contributing to the decline in population over the last four decades (Pérez-Flores, Arias-Domínguez & Arias-Domínguez, 2020). This mega-herbivore is classified as ‘endangered’ on the 2014 IUCN Red List of Threatened Species, with estimates suggesting approximately 4,500 individuals remaining in the field. Furthermore, populations of *T. bardii* are dwindling due to anthropogenic activities causing habitat loss and fragmentation that include, among others, urban development, pollution, over hunting, as well as local and global climate change (García et al., 2014).

Despite their endangered status, very few genetic and genomic resources exist for *Tapirus* in general, and a complete mitochondrial genome assembly and annotation does not exist for Baird’s tapir. An early study, using a fragment of the mitochondrial Cytochrome Oxidase subunit II (*cox2*) to examine the phylogenetic relationships among representatives of the genus *Tapirus*, suggested the divergence of three distinct *Tapirus* lineages (South American, Central American, and Asian) occurred 20–30 million years ago (Ashley, Norman & Stross, 1996). Two more recent studies that used DNA microsatellite markers in wild and captive *T. bairdii* populations revealed that *T. bairdii* was at increased risk of losing genetic variability due to inbreeding (Norton & Ashley, 2004a; Norton & Ashley, 2004b). Lastly, a recent study focusing on the phylogeography of *T. bairdii* and *T. pinchaque* reported a close phylogenetic relationship between *T. bairdii* and fossil tapir species from North America, supporting previously established phylogenetic relationships based on morphometric data (Ferrero & Noriega, 2007; Ruiz-García et al., 2012). Interestingly though, the molecular study of Ruiz-García et al. (2012) contradicts an earlier morphometric study by Hulbert (1995) that indicated a close relationship between *T. bairdii* and the South American tapir *T. terrestris*. A fifth species within *Tapirus* has been proposed and further genetic analysis may contribute to its confirmation (Cozzuol et al., 2013). Overall, the phylogenetic position of *T. bairdi* is still not completely resolved and might evolve as more phylogenetic data become available.

This study, for the first time, generated a genomic resource, for this species. Specifically, we focused on assembling and characterizing in detail the mitochondrial genome of *T. bairdii*. The information we generated was used to explore the phylogenetic position of *T. bairdii* among closely related species based on the protein coding genes (PCGs). Among others, we have analyzed nucleotide composition, codon usage, and selective constraints in PCG’s. Also, the secondary structure of tRNA genes and the control region was investigated in detail. Lastly, the phylogenetic position of *T. bairdii* amongst members of the Perissodactyla was examined using PCGs. The complete and detailed characterization of the mitochondrial genome is a stride towards improving understanding of the evolutionary relationships of *Tapirus bairdii*.

## Materials & Methods

### Collection, tissue sampling, and DNA genomic sequencing

We requested a blood sample fixed in ethanol (95%) from a specimen of *T. bardii* exhibited at Parque Xcaret, Playa del Carmen, Quintana Roo, Mexico. This sample was transported to the Laboratorio de Bioconservación y Manejo, Instituto Politécnico Nacional, Cuidad de Mexico, Mexico, for its posterior laboratory treatment. The Qiagen Blood and Tissue Kit (Qiagen, Hilden, Germany) was used in extraction of total genomic DNA from the tissue sample according to manufacturer’s instructions (Vivas-Toro, Ortega & Baeza, 2021). Following extraction, the Savannah River Ecology Laboratory at the University of Georgia, Aiken, performed next generation sequencing from the extracted DNA sample (Vivas-Toro, Ortega & Baeza, 2021).

An Illumina HiSeq sequencer (Illumina, San Diego, CA, USA) that used 2 × 200 cycle sequenced Illumina paired-end (PE) shotgun library (Vivas-Toro, Ortega & Baeza, 2021). The PE reads were prepared using standard protocol of the Nextera™ DNA Sample Prep Kit (Epicentre^®^). The pairs generated totaled 5,507,122 and were provided in FASTQ format by the facility (Vivas-Toro, Ortega & Baeza, 2021). DNA-seq data have been deposited in the NCBI Sequence Read Archive (SRA) under Bioproject ID: PRJNA785336, Biosample accession: SAMN23553527, and SRA accession: SRR17086167. Methods of extraction and sequencing the mitochondrial DNA followed standard protocol and has been utilized previously for other organisms (López-Cuamatzi, Ortega & Baeza, 2022; Vivas-Toro, Ortega & Baeza, 2021).

### Mitochondrial genome assembly

The mitochondrial genome of *T. bardii* was *de novo*-assembled using the pipeline GetOrganelle v1.6.4 (Jin et al., 2020). The mitochondrial genome of the congeneric *T. terrestris*, available in GenBank (KJ417810), was used as a reference. The run used k-mer sizes of 21, 55, 85, and 115.

### Annotation and analysis of the assembled mitochondrial genome

The newly assembled mitochondrial genome of *T. bairdii* was first annotated using the MITOS and MITOS2 web servers (http://mitos2.bioinf.uni-leipzig.de) (Bernt et al., 2013) with the vertebrate genetic code (code 2). Corrections to the start and stop codons were made using the server ExPASy (https://web.expasy.org) and the MEGAX software (Artimo et al., 2012). Visualization was performed using the CGView Server (beta) (http://cgview.ca/) (Stothard & Wishart, 2004) using the manually corrected annotation.

The codon usage and open reading frames of the protein-coding genes (PCG) were analyzed. The codon usage of the 13 PCG’s was predicted using the Codon Usage web server (https://www.bioinformatics.org/sms2) (Stothard, 2000) and visualized using the EZcodon tool in the web server EZmito (http://ezmito.unisi.it/ezcodon) (Cucini et al., 2021), both set to the vertebrate genetic code. tRNA genes were identified by MITOS and MITOS2, and the secondary structures were visualized in the Forna web server (http://rna.tbi.univie.ac.at/forna/) (Kerpedjiev, Hammer & Hofacker, 2015).

Selective pressure on the PCG’s was examined by comparing rates of nonsynonymous and synonymous substitutions. The values of K_A_ (number of nonsynonymous substitutions per nonsynonymous site: K_A_ = d_N_ = S_A_/L_A_), K_S_ (number of synonymous substitutions per synonymous site: K_S_ = d_S_ = S_S_/L_S_), and *ω* (K_A_/K_S_) were found for each PCG using the KaKs_calculator 2.0 (Wang et al., 2009; Wang et al., 2010; Conrad et al., 2021). The estimated *ω* values were based on a comparison between *T. bairdii* and its closely related species, *T. indicus* (KJ417810). The *γ*-MYN model was used in order to account for variable mutation rates across sites within each PCG sequence. When *ω* = 1, the PCG’s are assumed to be under neutral selection, for *ω* > 1 positive (diversifying) selection is assumed, and *ω* < 1 indicates negative selective constraints (purifying selection).

The long non-coding region, understood to be the control region, was analyzed. Repeats within the region were found using the Tandem Repeat Finder Version 4.09 web server (https://tandem.bu.edu/trf/trf.basic.submit.html) (Benson, 1999) and the BioPHP Microsatellite Repeats Finder web server (http://insilico.ehu.es/mini_tools/microsatellites/) (Bikandi et al., 2004). Manual comparison to known annotations of mammalian CR consensus sequences revealed conserved domains and blocks. The RNAstructure web server (https://rna.urmc.rochester.edu/RNAstructureWeb) provided predictions of secondary structures based on the lowest free energy (Bellaousov et al., 2013). A short non-coding region, understood to be the origin of replication of the light strand (O_L_), was also analyzed in the RNA-structure web server, with an attention to the presence of stem and loop structures.

### Phylogenetic position of *Tapirus bairdii*

Using PCGs, we examined the phylogenetic position of *T. bairdii* among other representatives of the order Perissodactyla and superfamily Tapiroidea. The newly assembled mitochondrial genome along with the mitochondrial genomes of 61 other specimens belonging to the Perissodactyla available in the GenBank database were used for the phylogenetic analysis conducted using the MitoPhAST V02 pipeline (Tan et al., 2015). Outgroups included three species belonging to the Artiodactyla (*Lama guanicoe*, *Vicugna vicugna*, and *Camelus ferus* [Fam. Camelidae]). MitoPhAST first extracted all 13 PCG nucleotide sequences from the species available in GenBank as well as from *T. bairdii*. Clustal Omega aligned the PCG nucleotide sequences after translation into amino acid sequences (Sievers & Higgins, 2014; Vivas-Toro, Ortega & Baeza, 2021). Poorly aligned regions were removed with trimAl (Capella-Gutiérrez, Silla-Martínez & Gabaldón, 2009) before the dataset was partitioned and the best fitting models of sequence evolution were selected with ProtTest (Abascal, Zardoya & Posada, 2005). Finally, the concatenated and partitioned PCG amino acid alignments were used to perform a maximum likelihood phylogenetic tree search in the software IQ-TREE (Nguyen et al., 2015). The robustness of the ML tree topology was ascertained by 1,000 bootstrap pseudoreplicates of the tree search.

## Results and Discussion

The pipeline GetOrganelle assembled the complete mitochondrial chromosome of *T. bairdii* with an average coverage of 562x (sequence available at GenBank (OM935749), also provided as File S1). The full mitochondrial genome of *T. bairdii* is 16,697 bp in length and contains 13 protein-coding genes (PCG’s), two ribosomal RNA genes (*rrnS* (12S ribosomal RNA) and *rrnL* (16S ribosomal RNA)), and 22 transfer RNA (tRNA) genes. All of the PCG’s are encoded on the heavy (H) or positive strand, excluding *nad6* which is found on the light (L) strand. Both ribosomal RNA genes and fifteen of the tRNA genes are also encoded on the L strand (Fig. 1, Table 1). The gene order, and distribution on each strand, is identical to that reported in the congeneric *Tapirus indicus* (Muangkram et al., 2015), as well as in the rhinoceros *Diceros bicornis* (Willerslev et al., 2009) and horses *Equus kiang* (Luo et al., 2011) and *Equus caballus* (Xu & Arnason, 1994), all members of Perissodactyla. Mitochondrial genome arrangements in mammals tend to remain stable, therefore the identical mitochondrial genome arrangement of *Tapirus* to members of Perissodactyla is expected, and exhibits the typical vertebrate arrangement (Boore, 2001).

**Table 1:** Mitochondrial genome of *Tapirus bairdii*. Arrangement and annotation.

Name	Type	Start	Stop	Strand	Size (bp)	Start Codon	Stop Codon	Anti-codon	Continuity
trnF(gaa)	tRNA	1	68	+	68			GAA	0
rrnS	rRNA	69	1038	+	970				0
trnV(tac)	tRNA	1039	1105	+	67			TAC	0
rrnL	rRNA	1106	2684	+	1,579				0
trnL2(taa)	tRNA	2685	2759	+	75			TAA	2
nad1	Coding	2762	3718	+	957	ATG	TAA		−1
trnI(gat)	tRNA	3718	3787	+	70			GAT	−3
trnQ(ttg)	tRNA	3785	3857	–	73			TTG	2
trnM(cat)	tRNA	3860	3928	+	69			CAT	0
nad2	Coding	3929	4970	+	1,042	ATA	T		0
trnW(tca)	tRNA	4971	5040	+	70			TCA	5
trnA(tgc)	tRNA	5046	5114	–	69			TGC	1
trnN(gtt)	tRNA	5116	5188	–	73			GTT	2
O _L_		5189	5221	+	33				−1
trnC(gca)	tRNA	5221	5286	–	66			GCA	0
trnY(gta)	tRNA	5287	5353	–	67			GTA	1
cox1	Coding	5355	6899	+	1,545	ATG	TAA		−3
trnS2(tga)	tRNA	6897	6965	–	69			TGA	7
trnD(gtc)	tRNA	6973	7039	+	67			GTC	0
cox2	Coding	7040	7723	+	684	ATG	TAA		3
trnK(ttt)	tRNA	7727	7793	+	67			TTT	1
atp8	Coding	7795	7998	+	204	ATG	TAA		−43
atp6	Coding	7956	8636	+	681	ATG	TAA		−1
cox3	Coding	8636	9419	+	784	ATG	T		0
trnG(tcc)	tRNA	9420	9488	+	69			TCC	0
nad3	Coding	9489	9834	+	346	ATA	T		1
trnR(tcg)	tRNA	9836	9903	+	68			TCG	0
nad4l	Coding	9904	10200	+	297	ATG	TAA		−7
nad4	Coding	10194	11571	+	1,378	ATG	T		0
trnH(gtg)	tRNA	11572	11640	+	69			GTG	0
trnS1(gct)	tRNA	11641	11699	+	59			TCT	0
trnL1(tag)	tRNA	11700	11769	+	70			TAG	0
nad5	Coding	11770	13590	+	1,821	ATA	TAA		−17
nad6	Coding	13574	14101	–	528	ATG	TAA		0
trnE(ttc)	tRNA	14102	14170	–	69			TTC	5
cob	Coding	14176	15315	+	1,140	ATG	AGA		0
trnT(tgt)	tRNA	15316	15384	+	69			TGT	0
trnP(tgg)	tRNA	15385	15450	–	66			TGG	0
CR		15451	16697	+	1,247				0

The mitogenome is compact with intergenic spaces and overlaps primarily between 1 and 7 bp, with two relatively long gene junctions at *atp8-atp6* (overlap = 43 bp) and *nad5-nad6* (overlap = 17 bp). A 1,247 bp long non-coding region was assumed to be the D-loop/Control Region (CR).

All 13 PCG’s in the mitogenome of *T. bairdii* exhibit the typical vertebrate mitochondrial start codons ATG (*n* = 10 PCGs) or ATA (*n* = 3 PCGs) (Table 1). Eight of the PCG’s end with the typical termination codon of TAA. The *cob* gene terminates with AGA, which is also identified to be a conventional mtDNA stop codon in vertebrates, including other representatives of the Perissodactyla: *T. indicus* (Muangkram et al., 2015) and *Diceros bicornis* (Willerslev et al., 2009). The other four genes (*nad2, cox3, nad3, nad4*) terminate with an incomplete stop codon T, as reported before in *T. indicus* (Muangkram et al., 2015) as well as in representatives of the sister clade Rhinocerotidae (Willerslev et al., 2009). Incomplete stop codons in mitochondrial genomes appear to be completed with A residues via post-transcriptional polyadenylation (Ojala, Montoya & Attardi, 1981).

**Figure 2:**
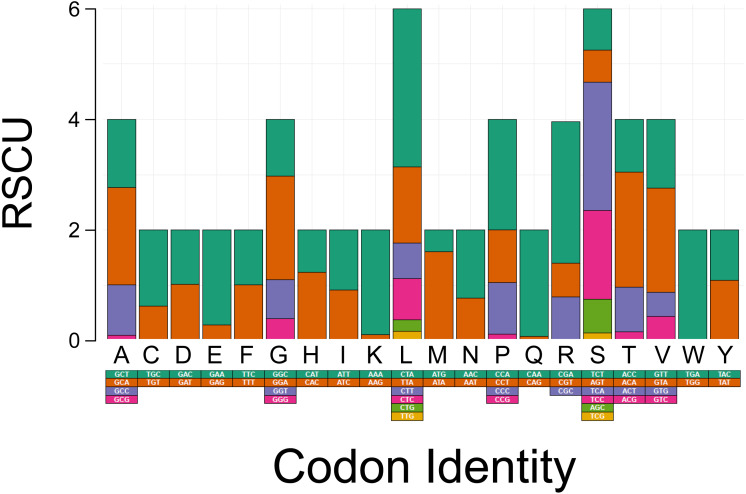
Codon usage analysis of the protein coding genes in the mitochondrial DNA of *Tapirus bairdii*.

The PCGs in *T. bardii* exhibit an A+T bias with an overall base composition of *A* = 32.2%, *T* = 29.8%, *C* = 25.6%, and *G* = 12.3%. This bias is also exhibited by other members of the Perissodactyla, including *T. indicus* (Muangkram et al., 2015) and *Equus kiang* (Luo et al., 2011). The most frequently used codons in the PCG’s of *T. bairdii* are CTA (Leu, *N* = 286 times used, 7.52% of total), ATA (Met, *N* = 203 times used, 5.34% of total), ATT (Ile, *N* = 180 times used, 4.74% of total), ACA (Thr, *N* = 169 times used, 4.45% of total), and ATC (Ile, *N* = 165 times used, 4.34% of total). The least frequently used codons are AGA (End, *N* = 1 time used, 0.03% of total), CGG (Arg, *N* = 1 time used, 0.03% of total), TGG (Trp, *N* = 2 times used, 0.05% of total), CAG (Gln, *N* = 4 times used, 0.11% of total), and TCG (Ser, *N* = 4 times used, 0.11% of total) (Fig. 2).

**Figure 3:**
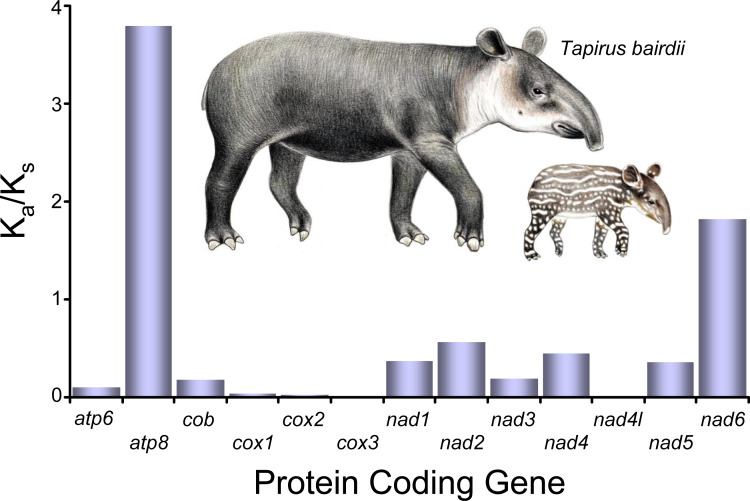
Selective pressure analysis in the PCG’s of *Tapirus bairdii*. K_A_/K_S_ (x10^−1^) values were calculated using the *γ*-MYN model. Illustrations of *Tapirus bairdii* copyright 1990 Stephen D. Nash. Used with permission.

All of the K_A_/K_s_ ratios for all of the 13 the PCG’s of *T. bairdii* show values <1, indicating all the PCG’s are under purifying selection (Fig. 3). The *atp8* K_A_/K_s_ ratio (K_A_/K_s_ = 0.3791) is much greater than that of the other 12 genes under purifying selection, suggesting a weaker constraint in the *atp8* gene. The K_A_/K_s_ ratios observed for *nad4l*, *cox1*, *cox2*, *cox3* (0.0000, 0.0037, 0.0027, 0.0016, respectively) were much lower in comparison, suggesting stronger purifying selection and evolutionary constraints in the aforementioned genes. Selective pressure analyses in mitochondrial PCG’s have not been conducted before in other species within the family Tapiridae and, in general, PCG selective pressures have been poorly studied in the Perissodactyla. A single previous study examining PCG’s of the mitochondrial genome in members of the genus *Equus* also found an overall pattern of purifying selection (Achilli et al., 2012).

**Figure 4:**
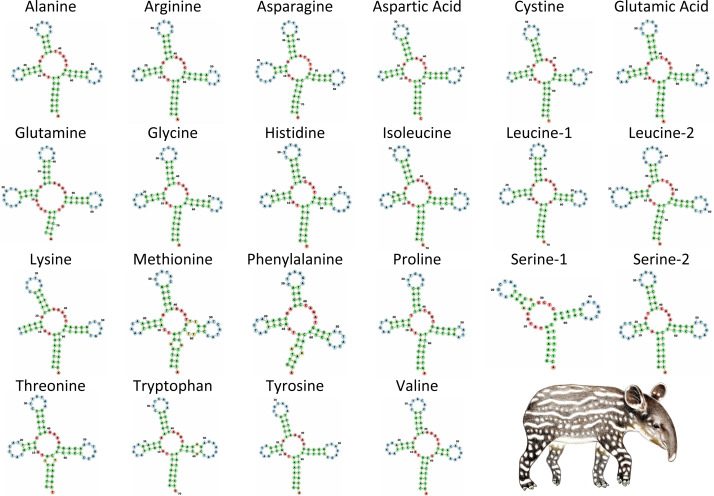
Secondary structure of tRNA’s in the mitochondrial genome of *Tapirus bairdii*. Secondary structure of tRNA’s visualized using the Forna web-server. Illustration of *Tapirus bairdii* copyright 1990 Stephen D. Nash. Used with permission.

The tRNA genes in *T. bairdii* range from 59 to 75 bp in length and all, except *trnS1*, exhibit the typical ‘cloverleaf’ secondary structure (Fig. 4). The *trnS1* gene was predicted to be missing the D-arm by MITFI (implemented in the MITOS software), similar to that reported before for the same tRNA gene in other members of the Perissodactyla, *i.e.*, *Tapirus terrestris* (Ashley, Norman & Stross, 1996) and *Rhinoceros unicornis* (Xu, Janke & Arnason, 1996). The loss of stem-loop structures, specifically in *trnS1*, is a common occurrence in almost all metazoan mitochondrial genomes (Watanabe, Suematsu & Ohtsuki, 2014), so the missing D-arm and shortened length of *trnS1* in. *T. bardii* is not unanticipated. Aminoacylation and EF-Tu binding of D-arm-lacking tRNAs have been identified as factors that assist in translation (Watanabe, Suematsu & Ohtsuki, 2014). The anticodon pattern is similar to that of members of Perissodactyla and that of the closely related Asian tapir (Muangkram et al., 2015), with the exception of *trnS1* codon which differs at the first position.

The *rrnS* (12s) and *rrnL* (16s) genes located on the heavy strand are 970 bp and 1,579 bp in length, respectively. The *rrnS* gene is located between the *trnF* and *trnA* genes, with a base composition of *A* = 38.6%, *T* = 23.1%, *C* = 22.0%, and *G* = 16.4%. The *rrnL* gene is located nearby between the *trnV* and *trnL2* genes, with a base composition of *A* = 38.1%, *T* = 24.5%, *C* = 21.6%, and *G* = 15.8%. Both genes show an A-T composition bias, as does the entire mitochondrial genome. Nucleotide composition analysis of the rRNA genes in other species of *Tapirus* has not been conducted. In the closely related *Rhinoceros unicornis*, a slight A-T bias was found for the *rrnL* gene, while a slight A-C bias was found for the *rrnS* gene (Xu, Janke & Arnason, 1996). The base composition for both rRNA genes of *R. unicoris* of thymine and cytosine was found to be between 21% and 25%, similar to the composition found in *T. bairdii*.

**Figure 5:**
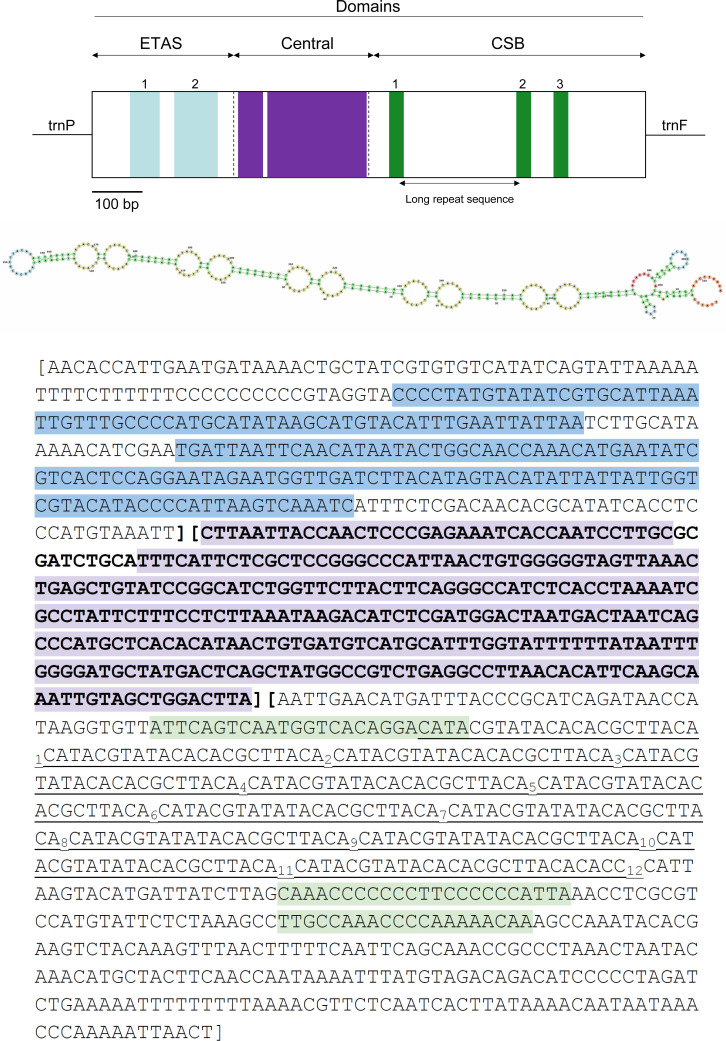
Visual representation of the control region (CR) in the mitochondrial genome of *Tapirus bairdii*. The CR is divided into the extended terminal association sequence (ETAS), central, and conserved sequence block (CSB) domains. Locations of the ETAS 1 and ETAS 2, CSB1, CSB2, CSB3 blocks, as well as the large highly conserved regions within the central domain are shown. The long repetitive motif is indicated in underline and a possible secondary structure is depicted for the region.

The full 1,247 bp long putative CR ranges from position 15,451 to 16,697 and is located between the *trnP* and *trnF* genes. The CR has a slight A-T skew, with an overall nucleotide composition of *A* = 33.2%, *T* = 25.6%, *C* = 28.7%, and *G* = 12.5%, which has been observed in other organisms (Muangkram et al., 2017). Stem-loop structures are located within this putative control region as well as microsatellite repeats. The microsatellite repeats-finder web server found 22 microsatellites within the CR most of them with AC or TA dinucleotide repeats (Table S1). A large tandem repeat 5′-(CAT ACG TAT ACA CAC GCT TAC A)_12_-3′ is found to begin at position 16,152 in the CR. The RNA-Structure web server produced 20 possible secondary structures all containing variable numbers and sizes of stem-loops throughout the entire sequence. These predictions ranged in values of Gibbs free energy (ΔG) from ΔG = −111.8 kcal/mol to ΔG = −111.4 kcal/mol (Fig. S1). The O_H_ region, within the control region (CR), is the origin of replication for the heavy strand (H-strand). The O_L_, origin of replication for the light strand (L-strand), of *T. bairdii* is a 33 bp long sequence with a stable stem-loop secondary structure and found within the WANCY cluster, similar to other vertebrate species (Seutin et al., 1994). A common characteristic for vertebrate mitochondrial genomes, the WANCY cluster, contains a series of five tRNA’s (*trnW*, *trnA*, *trnN*, *trnC*, and *trnY*) with a conserved order flanking the O_L_ region. Secondary structure prediction found 2 possible stem-loop secondary structures for the O_L_, ΔG = −11.9 kcal/mol and ΔG = 3.2 kcal/mol (Fig. S2).

The three functional domains of the control region found in mammals, namely the extended terminal association sequences (ETAS), central, and conserved sequence block (CSB) domains were also detected observed in the same region of *T. bairdii* (Fig. 5) (Sbisá et al., 1997). The length of each domain (ETAS = 322 bp, Central = 316 bp, CSB = 609 bp) is within the normal range of mammalian control regions, notably similar to that of *Tapirus indicus* (Sbisá et al., 1997; Muangkram et al., 2015). The CSB domain was A-T rich, with 56.3% A-T composition. The large tandem repeat found to be from position 16,152 to 16,420–between CSB-1 and CSB-2–is similar to others in Perissodactyla (Xu & Arnason, 1994; Muangkram et al., 2017). The function of the CSB is still unclear, but their occurrence in vertebrate mitogenomes in general suggests that they play a critical role in genome replication and transcription (Satoh et al., 2016). A high degree of conservation was observed in the Central domain between members of Perissodactyla and *T. bairdii* (after a multiple alignment of CR’s), which is expected amongst vertebrates (Sbisá et al., 1997). Conserved blocks within ETAS (1 and 2) and CSB (1–3) were also identified based on the alignment of sample Perissodactyla and comparison to other mammalian sequences (Sbisá et al., 1997). Higher nucleotide variability within the ETAS and CSB domains follows expectations due to higher substitution rates within these regions compared to the Central domain (Pesole et al., 1999). Further analysis is needed in the organization and annotation of the CR’s of Perissodactyla to better understand the variation between the species.

**Figure 6:**
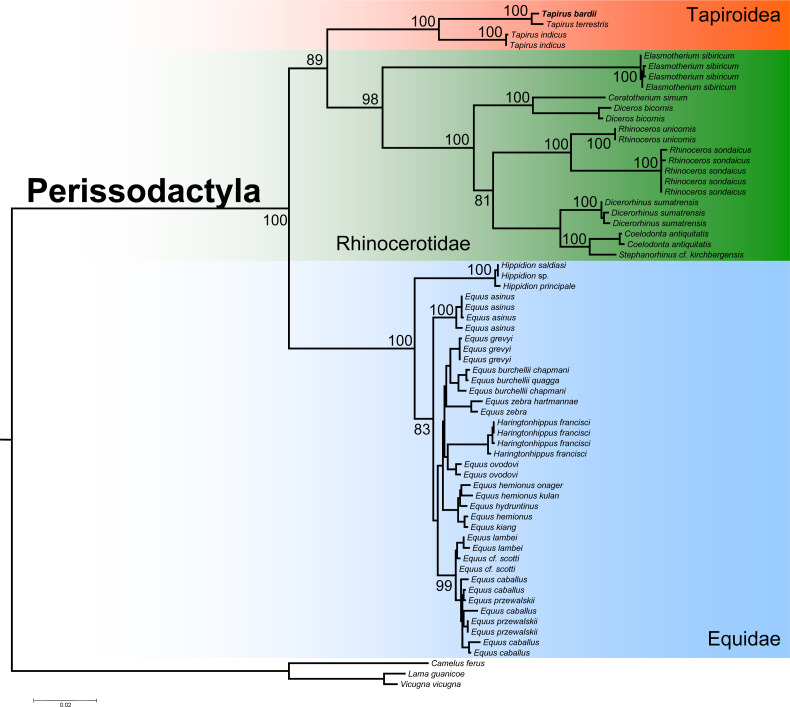
Phylogenetic analysis of *Tapirus bairdii* and related species in the order Perissodactyla. Total evidence phylogenetic tree obtained from ML analysis based on a concatenated alignment of amino acids of the 13 protein-coding genes present in the mitochondrial genome of representatives of the order Perissodactyla. Numbers above or below the branches represent bootstrap values.

The ML phylogenetic analysis confirmed the monophyly of the order Perissodactyla considering that all the species belonging to the superfamily Tapiroidea, including *T. bardii* and congeneric species, superfamily Rhinocerotidae, and family Equidae clustered into a single fully supported clade (Fig. 6). Within this monophyletic Perissodactyla, the family Equidae occupied a basal position, sister to a second clade composed of representatives from the families Tapiridae and Rhinocerotidae. Within the superfamily Tapiroidea, *T. bardii* was sister to *T. terrestris*. *Tapirus indicus* was sister to the fully supported *T. bardii* + *T. terrestris* clade. Ancestral mtDNA retrieved from fossil and/or subfossil specimens belonging to the genus *Tapirus* most likely will permit a deeper exploration of the phylogenetic relationships in these iconic mammals. The phylogenetic relationships among the different species of Rhinocerotidae are identical to those found by previous studies (Willerslev et al., 2009; Margaryan et al., 2020).

Given raising concerns about population decline experienced by multiple populations of *T. bairdii* across its range of distribution (Schank et al., 2020), it is fundamental to improve our understanding of population abundance, deme dynamics, as well as gaining knowledge on the presence/absence of this species in human altered habitats. Direct invasive sampling may not represent the optimal solution to understand the demography of *T. bairdii* considering this species has become elusive and given that invasive sampling can disrupt and or stress individuals and populations that may already be experiencing moderate or major local anthropocentric impact (Le Breton et al., 2019). We propose this newly assembled genome can be used as a reference for the retrieval (using bioinformatics strategies) of mitochondrial markers for bioprospecting and biomonitoring of *T. bairdii* when using indirect surveillance strategies such as environmental DNA (eDNA) in the form of scats or blood from insects (iDNA) (Schnell et al., 2015). Such efforts are currently being tested in other large herbivorous vertebrates with major conservation problems (*e.g.*, in moose) (Lyet et al., 2021) and we believe this study is a step forward towards to implementation of indirect surveillance in *T. bairdii*.

## Conclusions

This study assembled, for the first time, the full mitochondrial genome of the Central American Tapir, *T. bairdii.* This large proboscis-bearing mammal is threatened by deforestation and overhunting, contributing to population decline. The complete annotation and analysis of the mitochondrial genome will contribute to the understanding of selective pressures and evolutionary relationships of *T. bairdii* as well as providing more knowledge for use in conservation efforts of this iconic endangered mega-mammal from the Neotropics.

## Supplemental Information

10.7717/peerj.13440/supp-1 Supplemental Information 1 Sequence, mitochondrial genome Tapirus bairdii

10.7717/peerj.13440/supp-2 Supplemental Information 2 Secondary structure of the Control Region

10.7717/peerj.13440/supp-3 Supplemental Information 3 Secondary structure of the Origin of Replication in the Light Strand

10.7717/peerj.13440/supp-4 Supplemental Information 4 Position and identity of the microsatellites repeat in the Control Region

## Additional Information and Declarations

### Data Availability

The following information was supplied regarding data availability: The DNA-seq data are available at the NCBI Sequence Read Archive (SRA) under Bioproject: PRJNA785336, Biosample accession: SAMN23553527, and SRA accession: SRR17086167. The complete mitochondrial chromosome of *T. bairdii* is available at GenBank: OM935749.
