# Supplementary figures and images for: First genomic resource for an endangered neotropical mega-herbivore: the complete mitochondrial genome of the forest-dweller (Baird’s) tapir (Tapirus bairdii)
Source: PeerJ. 2022 Jun 1;10:e13440. doi: 10.7717/peerj.13440 (PMC9166683; doi:10.7717/peerj.13440)

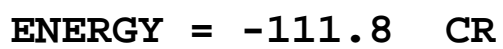

Supplement: Supplemental Information 2 [file peerj-10-13440-s002.pdf]
