# Supplementary material for: First genomic resource for an endangered neotropical mega-herbivore: the complete mitochondrial genome of the forest-dweller (Baird’s) tapir (Tapirus bairdii)
Source: PeerJ. 2022 Jun 1;10:e13440. doi: 10.7717/peerj.13440 (PMC9166683; doi:10.7717/peerj.13440)
Supplement: Supplemental Information 3 [file peerj-10-13440-s003.pdf]

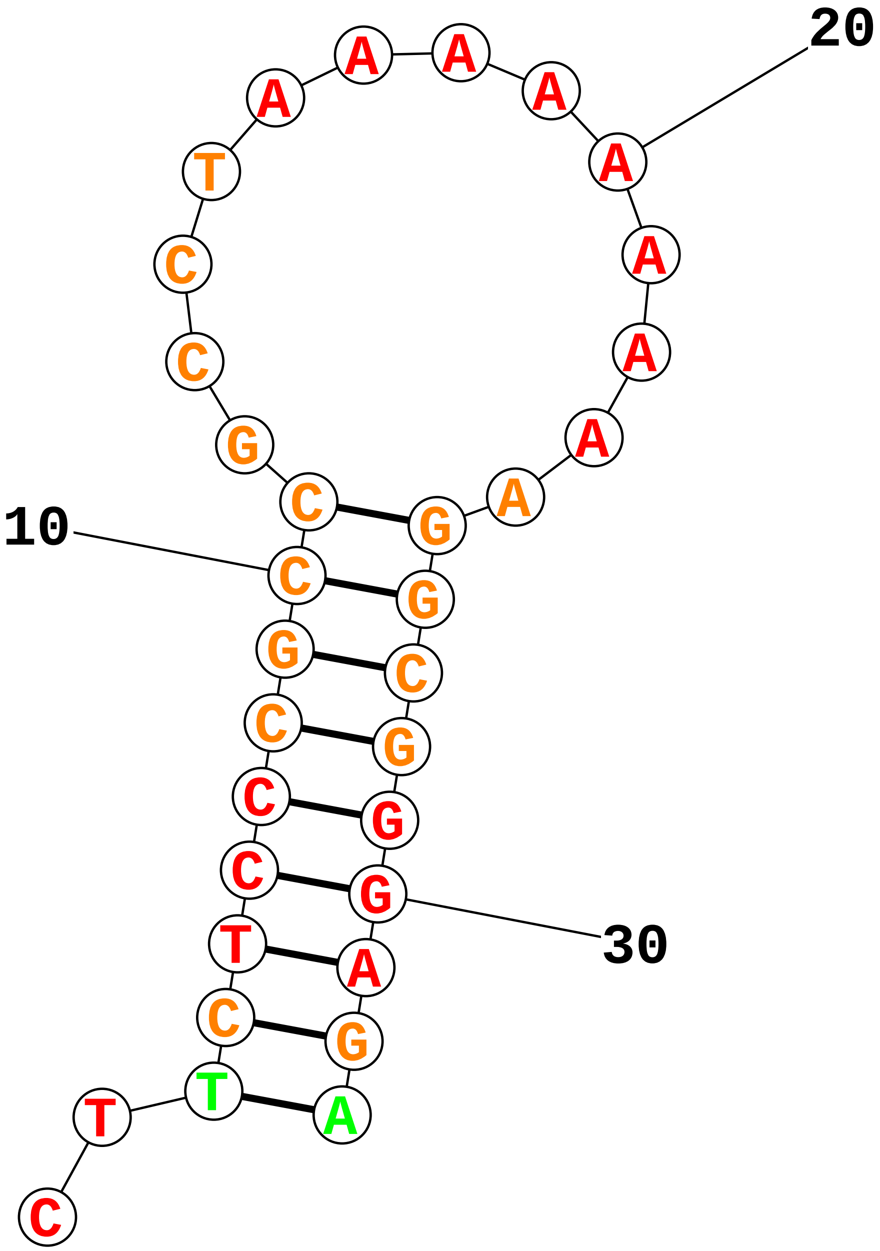

Probability >= 99%
99% > Probability >= 95%
95% > Probability >= 90%
90% > Probability >= 80%
80% > Probability >= 70%
70% > Probability >= 60%
60% > Probability >= 50%
50% > Probability

ENERGY = -11.9 OL
