# Supplementary material for: First genomic resource for an endangered neotropical mega-herbivore: the complete mitochondrial genome of the forest-dweller (Baird’s) tapir (Tapirus bairdii)
Source: PeerJ. 2022 Jun 1;10:e13440. doi: 10.7717/peerj.13440 (PMC9166683; doi:10.7717/peerj.13440)
Supplement: Supplemental Information 4 [file peerj-10-13440-s004.pdf]

Microsatellite Repeats Finder

Position	Cycle	Repeats	Sequence
28	2	3	GTGTGT
56	2	3	TTTTTT
62	3	3	CCCCCCCCC
246	3	3	TATTATTAT
525	2	3	CACACA
556	2	3	TTTTTT
710	2	3	ACACAC
732	2	3	ACACAC
754	2	3	ACACAC
776	2	3	ACACAC
798	2	3	ACACAC
820	2	3	ACACAC
839	2	3	TATATA
861	2	3	TATATA
883	2	3	TATATA
905	2	3	TATATA
927	2	3	TATATA
952	2	3	ACACAC
962	2	3	ACACAC
996	2	3	CCCCCC
1005	2	3	CCCCCC
1189	2	4	TTTTTTTT
